# Preparation of Polyvinyl Alcohol/Chitosan/*Antrodia cinnamomea* Polysaccharide Composite Film Incorporated with Tea Tree Essential Oil: Structure, Antioxidant, Antibacterial Activities, and Application in Postharvest ‘Yuluxiang’ Pear Preservation

**DOI:** 10.3390/foods15132300

**Published:** 2026-06-26

**Authors:** Wanhai Zhou, Yang Huang, Lu Chen, Anwar Noman, Ruizhang Feng, Yingmei Tao, Wanpeng Xi, Lianqing Hu, Wenwen Liu, Xianzhong Lv, Jinbo Chen, Mengyao Li

**Affiliations:** 1 Sichuan Oil Cinnamon Engineering Technology Research Center, Faculty of Agriculture, Forestry and Food Engineering, Yibin University, Yibin 644000, China; wanhaizhou@126.com (W.Z.); qq112023707042163@email.swu.edu.cn (Y.H.); 18381302395@163.com (L.C.); anwarnoman20@yibinu.edu.cn (A.N.); 2021030001@yibinu.edu.cn (L.H.); 2020060002@yibinu.edu.cn (W.L.); lxz160414@email.swu.edu.cn (X.L.); 13404050193@163.com (J.C.); 18090396165@163.com (M.L.); 2 College of Horticulture and Landscape Architecture, Southwest University, Chongqing 400716, China; 3 Key Lab of Aromatic Plant Resources Exploitation and Utilization in Sichuan Higher Education, Yibin University, Yibin 644000, China; 4 School of Food and Liquor Engineering, Sichuan University of Science and Engineering, Yibin 644000, China; ttkk051112@163.com

**Keywords:** *Antrodia cinnamomea* polysaccharide, tea tree essential oil, composite film, fruit preservation

## Abstract

Polyvinyl alcohol (PVA)/chitosan (CS)-based films incorporated with *Antrodia cinnamomea* polysaccharide (ACP) and tea tree essential oil (TTEO) were developed using a solution casting method. The physicochemical, bioactive, and structural attributes, as well as the effects of these films on post-harvest ‘Yuluxiang’ pears, were assessed. The results demonstrated strong interactions among all functional components. The integration of ACP reinforced the mechanical properties of PVA/CS-based films, whereas the combined incorporation of ACP/TTEO enhanced water resistance, ultraviolet-light shielding ability, and barrier performance against oxygen and water vapor. Contact angle measurements showed that the PVA/CS/ACP/TTEO composite film exhibited superior wettability and adhesion to pear surfaces. Furthermore, the PVA/CS/ACP/TTEO composite film exhibited potent antibacterial activity, recording 99.99% inhibition against *Staphylococcus aureus* and 99.91% against *Escherichia coli*. TGA and DTG analyses suggested that ACP improved the thermal stability and restricted the film’s degradation rate. Antioxidant assays revealed that the incorporation of ACP and TTEO markedly elevated the antioxidant ability of the PVA/CS-based film. After 21 days of storage, the PVA/CS/ACP/TTEO composite film effectively maintained firmness, titratable acidity, vitamin C levels, and the activities of superoxide dismutase and catalase in post-harvest pears. Moreover, the composite film delayed fruit yellowing and oiliness, lowered the accumulation of hydrogen peroxide and malondialdehyde, and significantly reduced microbial counts (*p* < 0.05). This study demonstrates that the fabricated PVA/CS/ACP/TTEO composite film possesses the ability to extend the shelf life of perishable fruits under ambient storage conditions.

## 1. Introduction

Food loss and waste have become a major global challenge. It is estimated that nearly one-third of global food production is lost or wasted annually, with fruits and vegetables representing approximately 45% [1]. Due to stringent appearance quality standards, the short storage life of fruits and vegetables, and numerous bottlenecks in storage, transportation, and packaging, a large quantity of fresh produce is discarded throughout the supply chain and at retail stages [2]. In this context, food packaging performs an irreplaceable function within the food industry, serving both to prolong food shelf life and to safeguard food safety. Nevertheless, beyond solid waste pollution, conventional plastic packaging poses dual threats to the environment and food safety. Considerable carbon emissions are released throughout production and incineration, accompanied by the migration of chemical contaminants into food [3]. Accordingly, modern manufacturers must balance market demands with ecological sustainability. The research and development of eco-friendly packaging technologies have become a top priority, with far-reaching implications for public health, economic development, and global ecological security [4]. Among various sustainable packaging approaches, active packaging is an innovative technique that can improve the quality and food safety of perishable foods while effectively prolonging their shelf life [5]. Active food packaging mainly consists of edible coatings (applied directly onto food) and external functional films [6]. Compared with edible coatings, functional films have a longer service life and self-healing performance, and can also improve the visual appearance of food. For these reasons, they have attracted increasing attention within food preservation [7].

In conventional film-forming materials, polysaccharides provide structural, protective, adhesive, and stimulus-responsive properties, and also exhibit antibacterial, anticancer, and antioxidant activities, making them key carbohydrate biopolymers [8]. Chitosan (CS) is extensively applied in active packaging to prolong the shelf life of various foods as films, coatings, and aerogels, owing to its outstanding film-forming ability, biodegradability, biocompatibility, and antibacterial activity [9]. However, CS generally suffers from a high hydrophilicity, poor gas barrier properties, insufficient antibacterial and antioxidant capacities, and a low mechanical strength. These drawbacks restrict its further application in food preservation [10]. To overcome these limitations, the properties of packaging materials need to be optimized by blending with other biopolymers, incorporating natural bioactive ingredients, constructing multilayer film structures, chemical modification, and adopting advanced processing technologies [11]. Among these approaches, blending with other biopolymers and incorporating natural bioactive ingredients are the most widely applied.

As a non-toxic, water-soluble, semi-crystalline polymer, polyvinyl alcohol (PVA) possesses superior film-forming performance, biocompatibility, light transmittance, and degradability [12]. Blending PVA with CS enables the formation of a stable composite network via intermolecular hydrogen bonds, which enhances the mechanical strength of the films and mitigates deformation resulting from CS’s strong hydrophilicity [13]. When blended with CS, PVA forms a stable composite network structure via intermolecular hydrogen bonds, which not only enhances the mechanical properties of the film but also reduces the film deformation induced by the high hydrophilicity of CS. Nevertheless, studies have demonstrated that PVA/CS composite films still have limitations, including a limited mechanical strength, poor thermal stability, and high moisture absorption [12]. Incorporating natural bioactive components into the PVA/CS matrix can endow the films with antioxidant and antibacterial properties, stabilize food quality, and inhibit microbial proliferation [14]. With broad-spectrum antibacterial, antifungal, and antioxidant properties, plant essential oils are widely employed as natural active agents in fruit and vegetable preservation and in controlling food spoilage caused by pathogenic bacteria [15].

Tea tree essential oil (TTEO) has gained considerable attention in the fabrication of active food packaging films owing to its remarkable antibacterial and antioxidant effects [16,17,18], as well as its ability to enhance the activities of defensive enzymes in fresh fruits and vegetables [19,20]. For instance, the composite film prepared from PVA/CS possesses excellent thermal stability, in vitro release performance, antioxidant activity, and antibacterial properties [21]. During composite film preparation, polysaccharides exert synergistic effects with essential oils and facilitate the homogeneous dispersion of essential oil molecules within the film matrix [22]. Other studies have indicated that the incorporation of TTEO tends to induce significant phase separation, which disrupts the internal microstructure of soybean polysaccharide/pullulan composite films and significantly deteriorates their overall physical properties [23]. Hence, the development of polysaccharide–essential oil composite materials for enhancing the functional properties of food packaging materials is of considerable scientific interest and practical significance.

*Antrodia camphorata* polysaccharide (ACP), extracted from *Antrodia camphorata* of the *Polyporaceae* family, exhibits prominent anti-inflammatory [24], anti-tumor [25], and antioxidant activities [26]. It contains abundant polar functional groups and presents excellent compatibility with CS and PVA. ACP can participate in forming a stable film network structure via hydrogen bonding and inhibit the rapid volatilization and loss of essential oils, thereby maintaining the long-term biological activity of composite films [27]. As a natural edible polysaccharide, ACP exhibits high safety with no risk of harmful substance migration. Its thickening and emulsifying properties can improve the homogeneity of film-forming solutions and mitigate phase separation in the solution system [28]. Accordingly, ACP holds great potential as a film-forming material for food preservation. However, current studies on ACP have mainly focused on basic medicine [29] and animal husbandry [30], while its applications in food preservation remain rarely reported. To the best of our knowledge, no studies have reported the fabrication of food fresh-keeping films by blending ACP with plant essential oils, PVA, and CS. Therefore, the feasibility of combining these functional materials to develop food fresh-keeping films and evaluate their preservation effects on fruits warrants further investigation.

Yuluxiang pear (*Pyrus sinkiangensis* ‘Yuluxiang’) is a major pear cultivar in northern China and is popular among consumers for its crisp flesh, abundant juice, and high sugar content [31]. Nevertheless, its extremely thin peel leads to prominent postharvest problems during storage. When transferred from refrigerated storage to ambient temperature, the fruit is prone to peel yellowing and surface greasiness development [32], which impair its visual quality, reduce its market competitiveness and commercial value, and eventually result in substantial economic losses for the fruit cultivation and distribution sectors. Previous studies have reported prolonging the shelf life of ‘Yuluxiang’ pear via exogenous melatonin [31], 1-methylcyclopropene [32], and ozone treatment [33]. However, none of these methods can simultaneously achieve the physical barrier, antioxidant, and antibacterial effects. Composite active packaging films fabricated from polysaccharides, essential oils, and other materials integrate the physical barrier, antioxidant, and antibacterial functions. They present great potential to effectively address a series of postharvest defects of ‘Yuluxiang’ pear, such as peel yellowing, surface stickiness, and rot after transfer from cold storage. To date, the preservation of ‘Yuluxiang’ pear using the combined application of TTEO and ACP has not been investigated.

Given the drawbacks of active packaging films incorporated with a single plant essential oil or a single polysaccharide alone, this study adopted edible ACP with distinctive physicochemical properties and bioactivities, as well as widely applied TTEO, as functional additives. Composite active packaging films were fabricated via the solution casting method using PVA/CS as film-forming matrices. The structure and comprehensive properties of the films were thoroughly characterized. Meanwhile, their antibacterial and antioxidant activities were evaluated, and the effects of PVA/CS/ACP/TTEO composite films on the quality and physiological indices of ‘Yuluxiang’ pear during storage were investigated. To our knowledge, this study is the first to incorporate ACP and TTEO into a PVA/CS composite film system. These findings offer a new avenue for the application of ACP in food active packaging and establish a theoretical basis for the practical use of this multi-component composite film in preserving perishable fruits.

## 2. Materials and Methods

### 2.1. Materials

Chitosan was sourced from Sinopharm Chemical Reagent Co., Ltd. (Shanghai, China), and polyvinyl alcohol from Chengdu Kolon Chemical Co., Ltd. (Chengdu, China). *Antrodia cinnamomea* polysaccharide (ACP, main constituent: β-glucan; purity: 70%; molecular weight: 52.7 kDa, monosaccharide composition and molar ratio: galactose: glucose: mannose: rhamnose = 1:7.15:1.08:0.28) was supplied by Shanxi Jiuzhou Kangyuan Biological Co., Ltd. (Yuncheng, China), and tea tree essential oil (TTEO; purity: 98%; density: 0.823 g/mL; principal constituent: terpinen-4-ol, 48.18%) was supplied by Sichuan Ailangte Biotechnology Co., Ltd. (Chengdu, China). Glycerol was obtained from Chengdu Kolon Chemical Co., Ltd. (Chengdu, China), Tween-80 from Beijing Lanjieke Technology Co., Ltd. (Beijing, China), and citric acid from Chengdu Jinshan Chemical Reagent Co., Ltd. (Chengdu, China). ‘Yuluxiang’ pears (eight-tenths ripeness) were purchased from fruit stores in Shanxi, China.

### 2.2. Fabrication of Composite Films

The PVA/CS film, PVA/CS/ACP film, and PVA/CS/ACP/TTEO composite films were fabricated separately via the solution casting procedure. Component dosages were screened based on preliminary experiments. The detailed procedure for preparing the optimized composite film was as follows: 0.37% ACP, 1.62% CS, and 0.38% PVA were added to a 2% citric acid solution, and the mixture was maintained in a water bath at 80 °C and stirred continuously with a magnetic stirrer (GL-3250B, Kylin-Bell, Nantong, China) until all raw materials were completely dissolved. Then, 1.78% glycerol was added as a plasticizer, followed by continued stirring for 10 min to ensure homogeneous dispersion. After the film-forming solution was naturally cooled to 40 °C, Tween-80 (0.25%) and TTEO (0.89%) were added sequentially, followed by stirring for 5 min. Subsequently, the film-forming solution was ultrasonicated for 5 min using an ultrasonic disperser (Scientz-1500F, Scientz, Ningbo, China). Next, the solution was subjected to centrifugation at 2000 rpm for 2 min using a centrifuge (ST4R Plus, Thermo Fisher Scientific, Bremen, Germany) to remove bubbles. After defoaming, the film-forming solution was cast onto polystyrene Petri dishes (13 cm × 13 cm) and gently shaken to spread evenly. Then, the Petri dishes were placed in an oven (101-2DB, TAISITE, Tianjin, China) and dried at 60 °C for 5 h until the films were fully formed. The films were subsequently transferred to an artificial climate chamber (RDN-1000B, DONGQI, Ningbo, China) maintained at 25 ± 2 °C and 55 ± 5% relative humidity for at least 48 h to reach a dynamic equilibrium with the ambient moisture content before further experiments.

### 2.3. Determination of Physicochemical Indicators of Composite Films

#### 2.3.1. Thickness

The film thickness was measured at four corners and one central point using a digital micrometer (IP65, Mitutoyo, Kanagawa, Japan). The mean value was calculated and documented as the film thickness, with the unit of mm. Each test was performed in triplicate.

#### 2.3.2. UV Blocking Performance and Opacity

The UV blocking performance of the films was assessed by an ultraviolet spectrophotometer (UH5300, Hitachi, Tokyo, Japan) to determine the UV light transmittance of the films at 315 and 340 nm. The film was cut into circular pieces with a diameter of 6 mm and then affixed to the inner surface of a 96-well microplate. The transmittance of the film was determined using a microplate reader (Multiskan SkyHigh, Thermo Fisher Scientific, Waltham, MA, USA) at 600 nm. Five parallel samples were set for each group, and all tests were performed three times. The opacity of the film was calculated using the following formula [34]:

(1) $$Opacity=\frac{A}{X}$$

where A represents the absorbance of the films at 600 nm; and X represents the film thickness (mm).

#### 2.3.3. Mechanical Properties

The films were cut into 60 mm × 10 mm strips according to the method of Xu et al. [35] with slight modification. A Universal Testing Machine (AGS-X 10KN, Shimadzu, Kyoto, Japan) was used to test the tensile strength (TS) and elongation at break (EAB) of the films at room temperature, using a crosshead speed of 60 mm/min and an initial grip distance of 40 mm. Each test was carried out in triplicate. The TS and EAB of the films were determined based on the following formulae:

(2) $$TS\;(MPa)=\frac{F}{b\times d}$$

(3) $$\mathrm{EAB}\;\left(\%\right)=\frac{L-L_{0}}{L_{0}}\times 100\%$$

where F represents the maximum tensile force endured by the sample at break (N), while b represents the sample thickness (mm), d represents the sample width (mm), L represents the sample length at break (mm), and L_0_ represents the initial sample length (mm).

#### 2.3.4. Color Difference

The brightness (L*), red–green value (a*), and yellow–blue value (b*) at five random positions of the film were measured using a Portable Colorimeter (CR-10 PLUS, Konica Minolta, Tokyo, Japan). First, the L*, a*, and b* values of the white calibration plate were measured as L1* = 92, a1* = −0.9, and b1* = −2.4. Each test was carried out in triplicate. The total color difference (ΔE) was determined according to the following formula:

(4) $$\Delta E=\sqrt{{\left(\Delta L^{*}\right)}^{2}+{\left(\Delta a^{*}\right)}^{2}+{\left(\Delta b^{*}\right)}^{2}}$$

#### 2.3.5. Water Content (WC), Swelling Degree (SD), and Water Solubility (WS)

A slightly modified procedure based on Ibeogu et al. [36] was used to measure these properties. Film samples (2 cm × 2 cm) were prepared, and their initial weight was recorded as m_0_. The samples were then oven-dried at 105 °C for 24 h and weighed as m_1_. Subsequently, the dried film was immersed in 30 mL of ultrapure water (25 °C) for 24 h. Surface moisture was removed using filter paper prior to weighing (m_2_). The films were then re-dried at 105 °C for 24 h and weighed again as m_3_. All measurements were performed in triplicate, and the properties were calculated as follows:

(5) $$WC\left(\%\right)=\frac{m_{0}-m_{1}}{m_{0}}\times 100$$

(6) $$SD\;(\%)=\frac{m_{2}-m_{1}}{m_{1}}\times 100$$

(7) $$WS\;(\%)=\frac{m_{1}-m_{3}}{m_{1}}\times 100$$

#### 2.3.6. Water Vapor Permeability (WVP) and Apparent Oxygen Permeability (AOP)

WVP was determined according to the procedure of Wei et al. [37]: different film samples were cut into uniform circular sheets without holes. Five points were randomly selected for thickness measurement, and the mean value was calculated. The circular sheet was used to seal a glass test cup (effective diameter 1.65 cm) containing 25 mL of deionized water. The cup was placed in a controlled incubator (25 °C and 50% relative humidity), and the weight was recorded every 12 h until the mass loss of water in the cup was constant. Each test was carried out in triplicate, and the properties were calculated as follows:

(8) $$WVP\;(g\cdot m^{-1}\cdot s^{-1}\cdot {\mathrm{Pa}}^{-1})=\frac{\Delta w\times L}{\Delta t\times A\times \Delta p}$$

where Δw is the mass loss of the test cup (g); L is the average film thickness (m); Δt is the duration of mass loss of the test cup (s); A is the permeation area of the film samples (m^2^); and Δp is the partial vapor pressure (Pa).

AOP was measured following the procedure of Chang et al. [38] with minor modifications: 2 mL of linoleic acid was added to a 100 mL conical flask (effective diameter 2.7 cm). The mouth of the conical flask was covered with the prepared film, secured tightly, and sealed with paraffin. The conical flask was incubated at 25 °C under a relative humidity below 5% and weighed daily for 7 days. Each experiment was carried out in triplicate, and the AOP was calculated based on the increase in the mass of the conical flask:

(9) $$AOP\;(mg\cdot {cm}^{-2}\cdot d^{-1})=\frac{\Delta m}{S\times t}$$

where AOP is the apparent oxygen permeability (mg·cm^−2^·d^−1^); Δm is the mass increase of the Erlenmeyer flask (mg); S is the film area (cm^2^); and t is the measurement time interval (d).

#### 2.3.7. Scanning Electron Microscopy (SEM) Observation of Microstructure

The films were cryo-fractured using liquid nitrogen. After gold sputtering treatment of the cross-sections, an Environmental Scanning Electron Microscope (Quattro S, Thermo Fisher Scientific, Waltham, MA, USA) was used to observe the microstructural morphology of the film cross-sections.

#### 2.3.8. Fourier-Transform Infrared Spectroscopy (FTIR)

After being ground into a powder, the samples were pressed into uniform pellets and analyzed using Fourier-Transform Infrared (FTIR) spectroscopy (Thermo Nicolet iS5, Thermo Fisher Scientific, Waltham, MA, USA). The scanning range was 4000–400 cm^−1^ with a resolution of 4 cm^−1^.

#### 2.3.9. X-Ray Diffraction (XRD) Analysis

The sample was dried to constant weight and subsequently analyzed by X-ray diffraction (XRD) (ULTIMA IV, Rigaku Corporation, Tokyo, Japan). XRD patterns were acquired at a scanning speed of 2°/min over a diffraction angle (2θ) range of 5–60°.

#### 2.3.10. Thermogravimetric Analysis (TGA)

Measurements were conducted using a Simultaneous Thermogravimetric Analyzer (STA300, Hitachi, Tokyo, Japan). The temperature range was 30–600 °C with a heating rate of 10 °C/min, and testing was performed under a nitrogen atmosphere.

#### 2.3.11. Water Contact Angle (WCA) Determination

The WCA of the film was measured using a Contact Angle Meter (LSA100, LAUDA Scientific, Lauda-Königshofen, Germany) to evaluate surface wettability. A 2 μL droplet of distilled water was dispensed onto the film surface by the instrument, and the image was immediately captured using a digital camera. The contact angle was measured using image analysis software (SurfaceMeter (TM) 1.2.2.6).

#### 2.3.12. DPPH Radical Scavenging Activity

Following the procedure described by Yang et al. [39], 1 mL of film solution was added to 3 mL of DPPH solution (0.15 mmol/L). The blank control was prepared by replacing the sample with an equal volume of distilled water, while maintaining identical conditions for the remaining steps. The mixture was reacted in the dark for 30 min, and the absorbance was recorded at 517 nm using an ultraviolet spectrophotometer. Each experiment was performed in triplicate, and the calculation formula is as follows:

(10) $$DPPH\;radical\;scavenging\;activity\;(\%)=\left(\frac{D_{0}-D_{1}}{D_{0}}\right)\times 100\%$$

where D_0_ and D_1_ represent the absorbance values of the blank control and sample groups, respectively, measured at 517 nm.

#### 2.3.13. ABTS Radical Scavenging Activity

ABTS radical scavenging activity was determined according to the procedure reported by Yang et al. [39]. ABTS was mixed with potassium persulfate and allowed to react in the dark for 12 h. The resulting solution was then diluted with absolute ethanol until an absorbance of approximately 0.7 at 734 nm was achieved, yielding the ABTS radical working solution. A 20 mg film sample was subsequently mixed with 4 mL of the ABTS working solution and incubated in the dark for 30 min. Absorbance was then recorded at 734 nm. Each experiment was performed in triplicate, and the calculation formula is as follows:

(11) $$ABTS\;radical\;scavenging\;activity\;(\%)=\left(\frac{A_{0}-A_{1}}{A_{0}}\right)\times 100\%$$

where A_0_ and A_1_ represent the absorbance values of the blank control and sample groups, respectively, measured at 734 nm.

#### 2.3.14. Antibacterial Activity Evaluation

The antibacterial activity of the films was evaluated against two bacterial strains, *Escherichia coli* (Gram-negative) and *Staphylococcus aureus* (Gram-positive), using the shake-flask method. The initial bacterial suspension was adjusted to approximately 10^8^ CFU/mL for both strains. A total of 0.20 g of UV-sterilized (2 h) film sample was immersed in 10 mL bacterial suspension, followed by shaking incubation at 37 °C and 180 rpm for 2 h. Three biological replicates were set for each treatment, alongside blank controls containing bacteria without film samples and sterile water blank for contamination monitoring. After incubation, the bacterial suspension was subjected to 10-fold serial dilution from 10^0^ to 10^−3^, and 200 μL of appropriate dilution was spread onto nutrient agar plates. Plates were incubated at 37 °C for 12 h, and colonies ranging from 30 to 300 CFU per plate were counted for calculation. The detection limit of this assay was 10 CFU/mL. The calculation formula is as follows:

(12) $$\mathrm{Antibacterial}\;\mathrm{rate}\;\left(\%\right)=\frac{C_{0}-C}{C_{0}}\times 100\%$$

where C_0_ and C represent the colony counts of the blank control and sample groups, respectively.

### 2.4. Determination of the Effect of Composite Films on ‘Yuluxiang’ Pear Preservation

#### 2.4.1. ‘Yuluxiang’ Pear Film Wrapping Treatment

A total of 150 ‘Yuluxiang’ pears of similar size, free of mechanical damage, pest infestation, and disease symptoms, were selected and randomly assigned to three groups. One group served as the untreated control, while the other two groups were wrapped in PVA/CS composite film and PVA/CS/ACP/TTEO composite film, respectively. The packaged fruits were then sealed and stored in an incubator at 20 ± 1 °C and 70–80% relative humidity for 21 days. Samples were collected at 7-day intervals to evaluate various fruit quality parameters under different treatments.

#### 2.4.2. Pear Peel Color Measurement

The fruit surface color was measured using a portable colorimeter according to the CIELAB color space (L*, a*, and b*). Measurements were taken at three random points on each fruit, and all experiments were conducted in triplicate.

#### 2.4.3. Weight Loss

Fruits in the three groups were weighed on days 0, 7, 14, and 21, with 10 fruits per group. All tests were performed in triplicate, and weight loss was obtained using the following formula:

(13) $$\mathrm{Weight}\;\mathrm{loss}\;\left(\%\right)=\frac{W_{0}-W_{f}}{W_{0}}\times 100\%$$

where W_0_ is the initial weight (g); and W_f_ is the final weight (g).

#### 2.4.4. Firmness

Three ‘Yuluxiang’ pears were randomly sampled from each treatment group, and a puncture test for firmness was performed at three uniform positions along the equatorial line of each fruit (without peeling). A durometer (GY-4, HANDPI, Yueqing, China) was applied perpendicularly to the fruit surface for the measurement, and the average firmness value was recorded in N. All measurements were carried out in triplicate.

#### 2.4.5. Acidity and Total Soluble Solids

The measurement was performed on the pulp at two symmetric positions along the fruit equator. The juice was squeezed out and analyzed using a handheld digital refractometer (PAL-BX/ACID12, Atago, Tokyo, Japan). The analysis was performed in triplicate.

#### 2.4.6. Vitamin C

According to the protocol reported by Fu et al. [40] with slight modifications, 10.00 g of pear sample was accurately weighed. A 2% acetic acid solution was added at a solid-to-liquid ratio of 1:2 (g:mL), and the mixture was homogenized. After incubation in the dark at room temperature (20 °C) for 30 min, the homogenate was centrifuged at 4000 r/min for 10 min. The resulting supernatant was collected and made up to 100 mL in a volumetric flask. The absorbance was recorded at 243 nm using a UV–Vis spectrophotometer. Each sample was analyzed in triplicate, and vitamin C content was determined from a standard calibration curve using the mean absorbance value:

(14) $$VC\;(mg/100\;g)=\frac{C\times V_{t}}{10\times m}$$

where c is the vitamin C concentration calculated from the standard curve (mg/mL); V_t_ is the total volumetric volume of the sample solution (mL); and m is the mass of the original sample (mg).

#### 2.4.7. Determination of Lignin, Hydrogen Peroxide (H_2_O_2_), and Malondialdehyde (MDA) Contents, and Activities of Polyphenol Oxidase (PPO), Superoxide Dismutase (SOD), and Catalase (CAT)

The contents of lignin, H_2_O_2_, and MDA, as well as the activities of PPO, SOD, and CAT in ‘Yuluxiang’ pears were obtained using commercial assay kits (Sangon Biotech., Shanghai, China), following the manufacturer’s protocol. The absorbance was measured using a microplate reader and a UV–Vis spectrophotometer. The analysis was performed in triplicate.

#### 2.4.8. Relative Conductivity Measurement

Six circular flesh discs (8 mm in diameter) were punched out and transferred to 20 mL of deionized water and shaken evenly. The initial conductivity was measured using a conductivity meter (DDS-307A, SINSPEC, Shanghai, China) and recorded as C_0_. After incubation at room temperature for 1 h, the conductivity was measured again and recorded as C_1_. The solution was then boiled in a water bath for 30 min, cooled to room temperature, and the final conductivity was recorded as C_2_. The analysis was performed in triplicate. The calculation formula is as follows:

(15) $$\mathrm{Relative}\;\mathrm{Conductivity}\;(\%)\;=\;(\frac{C_{1}-C_{0}}{C_{2}-C_{0}})\;\times \;100\%$$

#### 2.4.9. Total Microbial Count

The total plate count was determined in accordance with GB 4789.2-2022. National Food Safety Standard: Food Microbiological Examination-Aerobic Plate Count; State Ad-ministration for Market Regulation, Standardization Administration of China: Beijing, China, 2022 [41]. Mold and yeast counts were determined in accordance with GB 4789.15-2016. National Food Safety Standard: Food Microbiological Examination-Enumeration of Molds and Yeasts; Standardization Administration of China: Beijing, China, 2016 [42].

#### 2.4.10. Statistical Analysis

All experiments were performed with three replicates per group unless otherwise specified, and the data are presented as mean ± standard deviation (mean ± SD). Statistical analysis was conducted by one-way ANOVA followed by Duncan’s multiple-range test using, IBM SPSS Statistics for Windows, version 27.0 (IBMCorp., Armonk, NY, USA) and differences were considered statistically significant at *p* < 0.05.

## 3. Results and Discussion

### 3.1. Characterization of Composite Films

#### 3.1.1. FTIR Spectra

**Figure 1:**
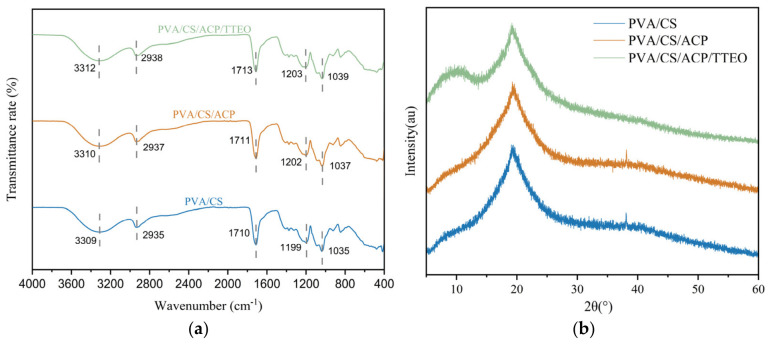
(**a**) Infrared spectra of films; and (**b**) XRD patterns of films.

The FTIR results of the various films are illustrated in Figure 1a. The absorption peaks of the films at 3309–3312 cm^−1^ were attributed to the stretching vibration of hydroxyl groups (-OH) in the PVA and CS structures. The peaks at 2935–2938 cm^−1^ were related to the asymmetric and symmetric stretching vibrations of -CH_2_- groups in the molecular structure [43]. These two peaks are typical characteristic asymmetric stretching peaks of PVA [44], and they also overlap with the symmetric and asymmetric stretching vibrations of -NH_2_ in CS [45]. The distinct band detected at 1710–1713 cm^−1^ may result from the C=O stretching vibration of acetate groups in PVA [46]. The infrared characteristic absorption peaks at 1199–1203 cm^−1^ and 1035–1039 cm^−1^ were assigned to the stretching vibration of the C-O-C ether bond. The intensity order of the characteristic peaks of the films was as follows: PVA/CS/ACP/TTEO > PVA/CS/ACP > PVA/CS. Regarding peak positions, compared with PVA/CS, the characteristic peaks of the other films all shifted toward higher wavenumbers, indicating that the incorporation of TTEO and ACP may enhance the vibrational coupling of these functional groups by optimizing intermolecular interactions. Castro et al. [44] also found that multiple characteristic peaks (especially those of hydroxyl groups) in the FTIR spectra of CS/PVA composite films incorporated with tea tree oil shifted to higher wavenumbers, which was assigned to the hydrogen bonding between hydroxyl groups in tea tree oil components (mainly 4-terpineol) and chitosan molecules. Notably, the incorporation of ACP and TTEO did not generate new characteristic peaks in the FTIR spectra of the films, implying that their incorporation did not disrupt the original chemical structures of CS and PVA, nor did it lead to the formation of new covalent bonds. This finding agrees with the report by Zhao et al. [47], who noted that, although the addition of essential oil did not generate new peaks in the films, it caused slight changes in the position and intensity of characteristic peaks, which might be caused by physical interactions among the components [48].

#### 3.1.2. XRD

XRD analysis was used to characterize the crystalline properties of the films in each group. As shown in Figure 1b, the PVA/CS film had a strong diffraction peak at 2θ = 19.8° and a weak characteristic diffraction peak at 38.1°, indicating that the film had a semi-crystalline structure. For the PVA/CS/ACP film, the peak intensity at 2θ = 19.8° decreased and the peak shape broadened, while the characteristic peak at 38.1° was further weakened, resulting in a 16% decrease in crystallinity. Zhao et al. [49] also found that intermolecular interactions formed between CS and Enoki mushroom foot polysaccharide restricted the molecular motion between them, leading to a decrease in crystallinity, causing changes in the mechanical and physical properties and forming a more stable composite structure, consistent with the present results. For the PVA/CS/ACP/TTEO film, the peak intensity at 2θ = 19.8° was further weakened, the peak at 38.1° almost disappeared, and a strong broad peak appeared at 2θ = 10°, forming crystal planes with larger d-values but a disordered arrangement, leading to a sharp 29% drop in crystallinity. These results indicate that the incorporation of TTEO into the film matrix reduced the peak intensity and made the structure more amorphous, which might be related to the plasticizing effect of TTEO [50]. Previous studies [51] also reported that the incorporation of TTEO into soluble soybean polysaccharide films did not significantly change the positions of diffraction peaks in the XRD patterns, although the peaks became broader and less intense compared with those of pure soluble soybean polysaccharide films.

#### 3.1.3. SEM

**Figure 2:**
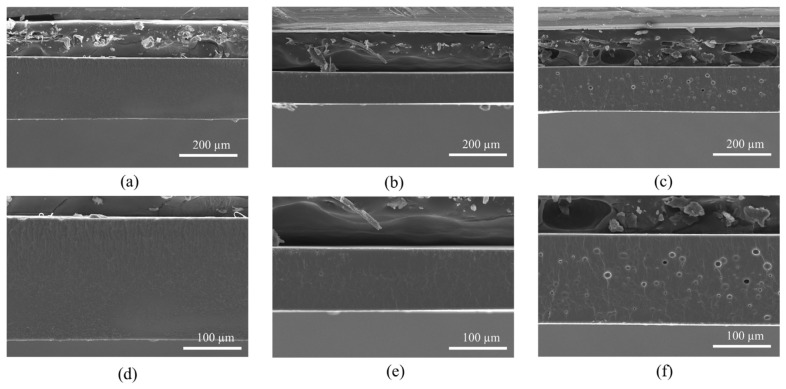
Cross-sectional SEM images of the films: (**a**) PVA/CS, 500×; (**b**) PVA/CS/ACP, 500×; (**c**) PVA/CS/ACP/TTEO, 500×; (**d**) PVA/CS, 1000×; (**e**) PVA/CS/ACP, 1000×; and (**f**) PVA/CS/ACP/TTEO, 1000×.

Figure 2 shows the cross-sectional SEM images of the three film groups after the fracture in liquid nitrogen and gold sputtering, revealing significant differences in the microstructure among the films. The cross-section of the PVA/CS film (Figure 2a,d) was relatively flat and smooth, and no obvious micropores were observed, indicating that a uniform and dense system was formed after blending PVA with CS. In contrast, the cross-section of the PVA/CS/ACP film (Figure 2b,e) showed a few stripes and micropores on one side, and this change in microstructure was consistent with the results of previous studies [52,53]. In addition, the cross-section of the PVA/CS/ACP/TTEO film (Figure 2c,f) showed an obvious aggregation of micropores and voids in its cross-section. This phenomenon may result from the migration and agglomeration of TTEO to the film surface during heating and evaporation in the drying process, eventually forming pores [51,54]. Sallam et al. [55] found that the addition of clove oil into the film also led to slight wrinkling on the film surface, reduced structural uniformity, and even partial phase separation due to the limited compatibility between the hydrophobic oil phase and the hydrophilic polymer matrix.

#### 3.1.4. TGA

**Figure 3:**
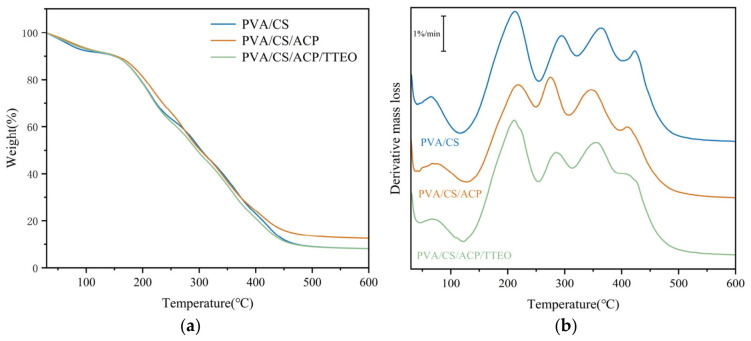
(**a**) Thermogravimetric analysis of films (TG); and (**b**) derivative thermogravimetry (DTG) curves of films.

The thermogravimetric (TG) and derivative thermogravimetric (DTG) curves are displayed in Figure 3a,b. In the temperature range of 30–112.82 °C, the mass loss of the films was mainly attributed to the evaporation of moisture inside the films. Among the samples, PVA/CS exhibited the highest weight loss (8.22%), whereas the PVA/CS/ACP showed the lowest (7.58%). These results indicate that PVA/CS had a relatively weak retention capacity for moisture and low-boiling-point substances, while the addition of ACP enhanced the film’s retention capacity for these substances to a certain extent.

In the temperature range of 112.82–247.74 °C, the weight loss of the three films increased significantly, which was mainly related to the volatilization of glycerol, the initial degradation of PVA and CS main chains, and the thermal decomposition of TTEO. The weight loss percentage of PVA/CS was 30.45%, and that of PVA/CS/ACP/TTEO was 32.09%. The slightly higher weight loss of the latter was attributed to the volatile compounds contained in TTEO [56]. The weight loss percentage of PVA/CS/ACP was 27.86%, which might be due to the presence of ACP stabilizing the polymer chains to a certain extent and slowing down the degradation rate of the film.

In the high-temperature range of 247.74–600 °C, this stage was mainly characterized by the deep degradation of residual polymer chains and the slow pyrolysis of inorganic components. The weight loss percentages of PVA/CS and PVA/CS/ACP/TTEO were 87.16% and 86.91%, respectively, while that of PVA/CS/ACP was 81.14%. It implies that the hydrogen bonding, physical interactions, and reinforcement between ACP and PVA/CS substrate can enhance the thermal resistance of the composite films. A similar study [57] found that thyme essential oil also caused a decrease in the crystallinity and thermal stability of curdlan/polyvinyl alcohol films.

#### 3.1.5. Appearance, Color Difference, and Opacity of the Films

**Figure 4:**
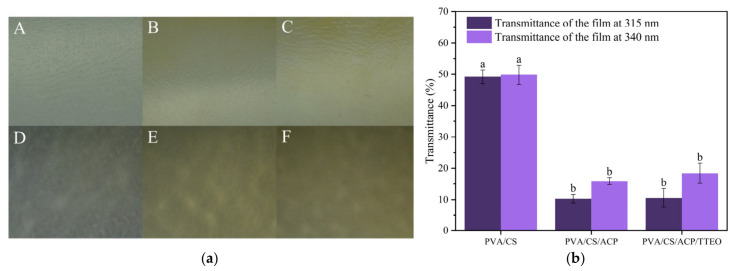
(**a**) Digital images of films: (**A**–**C**) PVA/CS, PVA/CS/ACP, and PVA/CS/ACP/TTEO (visual); (**D**–**F**) corresponding microscopic images; and (**b**) UV blocking performance. Different letters for the same index indicate significant differences among groups (*p* < 0.05).

Film color and opacity are important factors affecting the consumer adoption of film products [58,59]. As shown in Table 1, films with different compositions exhibited significant differences in color parameters and opacity. To complement the appearance analysis, Figure 4a directly presents the visual characteristics of the films. PVA/CS was transparent and colorless, while PVA/CS/ACP and PVA/CS/ACP/TTEO appeared yellowish-brown. The transparency of films is affected by the inherent color of the added materials [60]. In this study, ACP was a dark brown powder. After adding 0.37% ACP into the system, the opacity of PVA/CS/ACP increased significantly, the L* value decreased remarkably, and the a*, b*, and ΔE values increased notably. These results indicate that ACP reduced the film’s lightness and enhanced both opacity and the yellow hue, consistent with the findings reported by Liu et al. [61]. They observed comparable changes in opacity and color difference after incorporating *Pleurotus ostreatus* polysaccharides into CS films. After the addition of TTEO, no significant alterations were detected in the L* and ΔE values of the PVA/CS/ACP/TTEO film; however, the a* and b* values decreased to some extent, weakening the yellow hue caused by ACP. Combined with the SEM results, it was hypothesized that the increased voids and micropores in the film structure induced by the essential oil may alter the optical properties of the film.

#### 3.1.6. UV Blocking Performance

Maintaining food quality largely depends on protection against ultraviolet radiation [62]. Protecting food from light exposure, especially UV radiation, is one of the key functions of food packaging materials [63]. The UV barrier performance of different films is shown in Figure 4b. The UV transmittance of PVA/CS/ACP at 315 nm and 340 nm was significantly lower compared to that of the PVA/CS film. This difference may be attributed to the increased film thickness caused by the polysaccharides within the film, which reduces UV transmittance through the matrix [49]. In addition, the abundant functional groups in CS polysaccharide molecules, such as hydroxyl and carbonyl groups, can enhance UV barrier performance by absorbing UV light [64]. After the addition of TTEO, the UV transmittance of the film increased slightly, which may result from the increased voids and micropores formed in the film due to TTEO, thereby affecting the optical parameters of the film. However, the film still maintained a strong overall UV barrier capacity. It is worth noting that improvements in the UV barrier ability are typically accompanied by a reduced transparency [65], which is in agreement with the data shown in Table 1.

#### 3.1.7. Mechanical Properties and Thickness

Mechanical properties of the films serve as a crucial criterion for assessing their suitability as packaging materials [66]. Among them, TS and EAB are the most important mechanical properties. As illustrated in Table 2, the incorporation of ACP significantly increased the TS of the PVA/CS-based film. A similar phenomenon was also reported in a previous study [67], where *Lycium barbarum* polysaccharide improved the TS and EAB of starch films. The addition of TTEO slightly increased the TS of the film to 4.13 ± 0.05 MPa; however, the change was not significant. Liu et al. [68] and Fallah et al. [69] observed comparable effects in soluble soybean polysaccharide-based films and gelatin-based films, respectively. In contrast, the EAB of the film increased significantly to 392.21 ± 3.12% (*p* < 0.05) upon the addition of ACP, indicating that ACP enhanced film flexibility. Subsequently, the introduction of TTEO decreased EAB to 382.22 ± 4.06%. A previous study [48] also reported that increasing the content of *Litsea cubeba* essential oil resulted in a decrease in the EAB of the films.

Thickness is a basic parameter for evaluating the physical properties of films, which affects the color, transparency, permeability, and mechanical properties [57,70]. As shown in Table 2, the thicknesses of the three films did not differ significantly (*p* > 0.05) and were maintained in the range of 0.15–0.155 mm. This suggests that the incorporation of ACP and TTEO had no significant effect on film thickness, likely because of their low loadings. In similar studies, Hao et al. [71] reported no significant thickness differences among films prepared with different concentrations of *Gelidium amansii* polysaccharides. In addition, Liu et al. [61] found that *Pleurotus ostreatus* polysaccharide did not significantly alter the thickness of CS films, although the TS and EAB changed markedly.

#### 3.1.8. WC, SD, WS, WVP, and AOP

As displayed in Table 2, PVA/CS demonstrated the highest WC and SD. The incorporation of ACP and TTEO gradually reduced the WC, indicating that they occupied hydroxyl groups on CS and PVA through hydrogen bonding, thereby limiting the interaction between water molecules and the film matrix and ultimately decreasing the films’ water solubility [72]. The significantly reduced SD of PVA/CS/ACP suggests that ACP increased the cross-linking density of the film matrix and restricted water penetration. These results are in agreement with a previous study [73], in which mulberry leaf polysaccharides added to carboxymethyl cellulose-based films bound to matrix molecules via hydrogen bonds, inhibiting hydrophilic groups and thereby reducing SD. PVA/CS/ACP/TTEO showed the lowest SD, which may be attributed to the inability of non-polar components in TTEO to interact with water, resulting in a decrease in the film SD [74]. The reduction in SD is an important characteristic for inhibiting water diffusion into dense composite films, indicating the enhanced interaction ability of the composite materials [67].

Among all the films, PVA/CS exhibited the lowest WS (7.60 ± 0.36%). Following the incorporation of ACP, the WS of the film increased significantly to 41.26 ± 3.64%, a finding consistent with previously reported results [75]. The hydrophilic nature of polysaccharides might be one of the reasons for the high WS [76], since the hydrophilic groups in polysaccharides can interact with water, thereby increasing the film solubility [77]. In contrast, the incorporation of TTEO did not significantly influence the WS of the films. Overall, the incorporation of ACP and TTEO led to a decrease in SD and a simultaneous increase in WS of the films, suggesting that the composite film matrix possessed a stable structure and inherent biodegradability, and could tolerate high-humidity environments [78], and no film hydrolysis or rupture was observed under the incubator conditions for pear preservation.

Compared with PVA/CS, PVA/CS/ACP showed a significantly increased WVP, likely due to the hydrophilicity of ACP, which promotes water vapor transmission, as well as structural defects in the film that also increase WVP [79]. The WVP of PVA/CS/ACP/TTEO was significantly lower than that of the other films, indicating that, although the incorporation of TTEO induced more micropores and voids in the film, the essential oil itself was a non-polar substance with a certain hydrophobicity [80]. Yu et al. [81] observed that cinnamon essential oil improved the water barrier performance of chitosan/polyvinyl alcohol/hydroxypropyl methylcellulose/alizarin composite films, which was attributed to the formation of hydrogen bonds between the additive and the film matrix, thus hindering water molecule penetration.

Consistent with the variation trend of WVP, the AOP was the highest for PVA/CS/ACP and lowest for PVA/CS/ACP/TTEO. This may be because ACP increased the matrix porosity and facilitated oxygen diffusion, whereas the strong antioxidant and oxygen-scavenging capacities of TTEO increased the resistance to gas passage and reduced the AOP value [82].

#### 3.1.9. WCA

**Figure 5:**
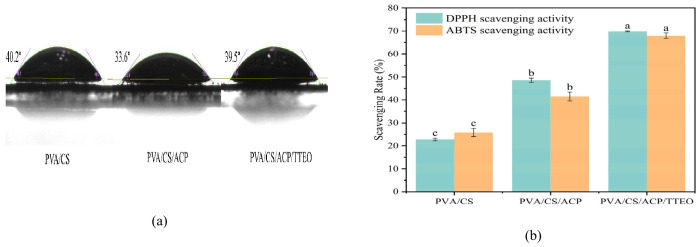
(**a**) Water contact angle images of films; and (**b**) antioxidant activity of films. Different letters for the same index indicate significant differences among groups (*p* < 0.05).

WCA is an important indicator for evaluating the water resistance, wettability, and adhesion of films. Figure 5a illustrates the WCA on the surface of different films. After the addition of ACP into the system, the WCA of the film decreased sharply to 33.6°, which may be assigned to the inherent hydrophilicity of ACP. Similar results were reported by other researchers [83], who observed a decrease in the WCA of gelatin-based films after adding rice bran polysaccharide. The incorporation of TTEO increased the WCA of the film to 39.5°, indicating that TTEO improved the hydrophobicity of the film. Bu et al. [11] found that TTEO contributed significantly to the hydrophobicity of films, as the hydrophobic groups in TTEO reduced the availability of free hydroxyl groups on the film surface through synergistic interactions, thereby improving hydrophobicity. Overall, all three films were hydrophilic films (θ < 65°), and this high hydrophilicity might be caused by the polar -NH_2_ groups of CS and -OH groups of PVA [76]. Notably, a smaller contact angle corresponds to greater wettability and adhesion [84]. This high wettability and adhesion were also observed during application, allowing the film to wrap the surface of pears effectively and tightly.

#### 3.1.10. Antioxidant Activity

Oxidation directly damages the nutritional components, flavor, and appearance of food, posing a significant threat to its quality and commercial value [85]. As shown in Figure 5b, the antioxidant activities of the three films increased significantly. Among them, PVA/CS showed the lowest DPPH and ABTS radical scavenging rates, which were 22.76 ± 0.62% and 25.85 ± 1.86%, respectively. The DPPH scavenging activity of this film might be related to the glycerol contained in it, as glycerol could act as an electron donor to scavenge DPPH molecules or exert an effect through plasticization [21]. After the addition of ACP, the DPPH and ABTS radical scavenging rates of PVA/CS/ACP significantly increased to 48.63 ± 0.95% and 41.44 ± 1.96%, respectively. This was because ACP possessed certain antioxidant activity, and a similar study [86] found that adding *Auricularia auricula* polysaccharide to CS films increased the DPPH scavenging rate of the films. Additionally, after the further introduction of TTEO, the scavenging rates reached the highest values of 69.88 ± 0.25% and 67.99 ± 1.20%, respectively. This was due to the inherent antioxidant properties of TTEO, which could enhance the DPPH [87] and ABTS radical scavenging rates of the films [88], consistent with previous reports [89].

#### 3.1.11. Antibacterial Properties

**Figure 6:**
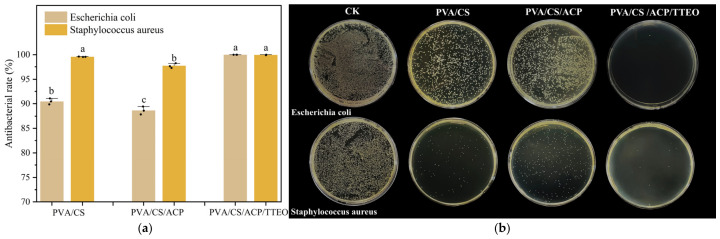
(**a**) Antibacterial rate of films; and (**b**) antibacterial effect of films. Different letters for the same index indicate significant differences among groups (*p* < 0.05).

*Escherichia coli* and *Staphylococcus aureus* are common pathogenic bacteria responsible for foodborne diseases over the past two decades [90]. The antibacterial activities of the three films against the tested bacterial strains were tested by the shake flask method. As shown in Figure 6a,b, all films exhibited antibacterial activity, and the antibacterial effect was jointly affected by the film composition and strain type. Among them, PVA/CS showed certain antibacterial activity against both bacteria, and the core reason was the excellent antibacterial property of CS [91]. Although the antibacterial mechanism of CS has not been fully clarified, previous studies [92] have suggested that the positive charge of protonated amino groups in CS interacts with negatively charged molecules on the bacterial cell surface, resulting in the permeabilization of the bacterial cell membrane and leakage of intracellular substances, thus culminating in the death of the bacterial cells. Both the PVA/CS and PVA/CS/ACP films exhibited greater antibacterial activity against *Staphylococcus aureus* than against *Escherichia coli*. This difference is primarily attributed to the enhanced susceptibility of Gram-positive bacteria to the bactericidal action of chitosan compared to Gram-negative strains [93]. Interestingly, PVA/CS/ACP exhibited the weakest antibacterial effect, possibly because ACP reduced the antibacterial activity of CS. In contrast, the antibacterial efficacy of the film was significantly enhanced after the introduction of TTEO. On the one hand, previous studies have reported that terpinen-4-ol, one of the major bioactive constituents of the essential oil, contributes substantially to its antibacterial activity by damaging bacterial biofilms, disrupting the lipid structure of the cell membrane, causing the leakage of intracellular constituents, and ultimately leading to microbial death [94,95]. On the other hand, as the core antibacterial material in the film, TTEO can exhibit an antibacterial effect on bacteria in synergy with CS [96]. Previous studies [97] have confirmed by the disk diffusion method that TTEO can significantly enhance the antibacterial efficacy of PVA-based films.

### 3.2. Application of Composite Films for ‘Yuluxiang’ Pear Preservation

#### 3.2.1. Pear Peel Color Difference

**Figure 7:**
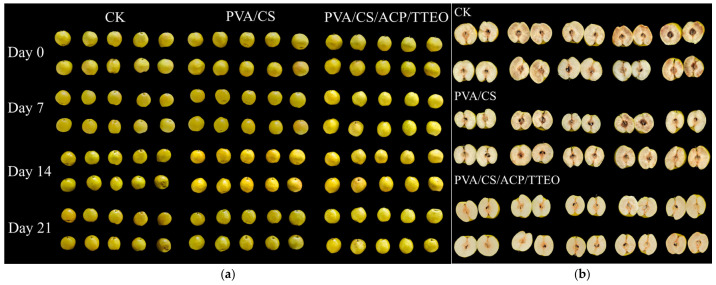
(**a**) Changes in appearance of ‘Yuluxiang’ pears during 21 days of shelf life; and (**b**) longitudinal section images of ‘Yuluxiang’ pears at 21 days of shelf life.

Previous studies [98] have established L* as an additional physiological indicator that effectively reflects the greasiness level of ‘Yuluxiang’ pears, and a higher L* value corresponds to a higher degree of greasiness in the fruit. As shown in Table 3, the color parameters of the control group (CK) increased more markedly compared with the other groups, indicating that the fruit without film protection suffered from excessive greasiness accumulation due to vigorous metabolism and severe color deterioration. Meanwhile, Figure 7a shows the changes in appearance of ‘Yuluxiang’ pears during 21 days of shelf life, and Figure 7b presents longitudinal section images of ‘Yuluxiang’ pears at 21 days of shelf life.

As shown in Figure 7a, the CK group exhibited obvious yellowing over the storage period, accompanied by visible water loss and shriveling on the epidermis of some fruits. Notably, the PVA/CS film could delay the changes in a* and b* values via a certain barrier effect, but its L* value reached the maximum on 7 days and then decreased gradually, which might be attributed to pericarp browning caused by premature ripening [99]. In contrast, the variation in color parameters for the PVA/CS/ACP/TTEO treatment group was minimal, indicating that this film effectively reduced greasiness migration and pigment degradation in the pericarp.

#### 3.2.2. Fruit Quality Parameters

Pears are classified as climacteric fruits, characterized by a rapid postharvest softening process that serves as a hallmark of senescence; this physiological progression significantly curtails shelf life, exacerbates postharvest decay, and presents a major challenge for storage and transportation [100,101]. As shown in Table 4, the firmness of pears in all groups exhibited a gradual decrease during shelf life. Compared with the CK group, the PVA/CS group showed significantly higher firmness at 7 days, with no significant differences at other time points. In contrast, the PVA/CS/ACP/TTEO group maintained the highest firmness throughout the shelf life, which might be attributed to the film’s ability to inhibit fruit metabolism and delay the reduction in firmness due to its low AOP value. This was consistent with a previous finding [85] that treatment with propolis ethanol extract/guar gum composite film could significantly reduce firmness loss in “Nanguo” pears.

As illustrated in Table 4, the TSS content of pears increased continuously during their shelf life, which was mainly derived from the hydrolysis of starch or cell wall polysaccharides stored in the fruit [102]. As presented in Table 2, after 7 days of storage, the CK group exhibited a significantly higher TSS content than the other groups, which may be associated with its higher weight loss rate. No significant difference in TSS content was observed among all groups after 7 days. The TA content of pears decreased continuously during their shelf life, and its change was affected by the metabolic rate, particularly the respiration rate, which consumed organic acids and might also lead to fruit senescence [99]. The TA content of the CK group decreased the most significantly, while the PVA/CS/ACP/TTEO group retained the highest TA content. This was in accordance with a previous study [103], which found that chitosan/pullulan/zein-based films incorporated with *Artemisia argyi* essential oil could reduce the TA consumption of *Phyllanthus emblica* fruits. The above results might be due to the low gas permeability of the films, which slowed down fruit respiration and required a longer time to synthesize and utilize metabolites, thereby suppressing the increase in TSS and the degradation of TA [104].

Vitamin C degradation during storage is mainly attributed to microbial infection, external oxidation, and ongoing physiological metabolic activity [105]. The vitamin C content in all treatment groups decreased with the progression of shelf life. On 7 days, the vitamin C content of the PVA/CS/ACP/TTEO group was significantly higher than that of the other groups, and this group exhibited the highest vitamin C retention throughout the shelf life, which might be associated with the strong antioxidant activity of ACP and TTEO. Previous studies [106] found that CS-based films incorporated with daisy essential oil could reduce vitamin C degradation in kiwifruit.

The weight loss rate is widely recognized as a key indicator closely associated with fruit senescence [107]. Compared with the CK group, the PVA/CS and PVA/CS/ACP/TTEO groups significantly reduced the weight loss rate of pears at 7 days, and the latter exhibited the lowest weight loss rate at 21 days, indicating that it could more effectively reduce fruit water loss and weight loss. Similarly, a related study found that CS/pullulan films incorporated with thyme essential oil nanoemulsion could reduce the weight loss rate of strawberries [108].

#### 3.2.3. Activities of PPO, CAT, and SOD

**Figure 8:**
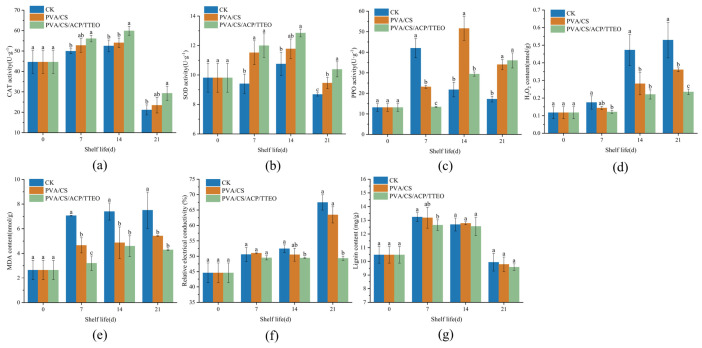
Influence of different treatments on the physiological indices of ‘Yuluxiang’ pear fruits during shelf life: (**a**) CAT activity; (**b**) SOD activity; (**c**) H_2_O_2_ content; (**d**) MDA content; (**e**) PPO activity; (**f**) relative electrical conductivity; and (**g**) lignin content. Different letters assigned to the same index at each 7-day sampling point indicate significant differences among groups (*p* < 0.05).

Antioxidant enzymes, such as SOD and CAT, play essential roles scavenging reactive oxygen species (ROS) and maintaining cellular homeostasis [109]. SOD can promote the dismutation of superoxide anions generated during the plant oxidative burst into H_2_O_2_, and CAT can alleviate the excessive accumulation of H_2_O_2_ in plants, thereby preventing cellular damage [110]. As illustrated in Figure 8a,b, the activities of SOD and CAT in all groups increased, reaching a peak at 14 days, and subsequently declined. Combined with Figure 8c, it can be inferred that this might be due to the accumulation of ROS exceeding the capacity of the antioxidant system during fruit senescence, which damaged the enzyme structure. During storage, the activities of SOD and CAT in the PVA/CS group were slightly higher than those observed in the CK group, but the difference was not significant; the PVA/CS/ACP/TTEO group maintained high activities of both enzymes throughout, which might be attributed to the antioxidant ability of ACP and TTEO that alleviated the inhibition of SOD by oxidative stress and stimulated the fruit’s own antioxidant system.

During storage, fruit senescence, the degradation of pectin substances in the cell wall, and microbial invasion can cause subcellular separation, damage membrane integrity, and allow oxygen penetration, thereby increasing the activity of PPO, which is responsible for phenol oxidation [111]. As PPO is closely related to fruit browning, Figure 7a shows alterations in the appearance of ‘Yuluxiang’ pears during 21 days of shelf life, and Figure 7b shows longitudinal section images of ‘Yuluxiang’ pears at 21 days of storage period.

The browning degree of the PVA/CS/ACP/TTEO group was the lowest, while the other two groups exhibited relatively severe browning. This result was consistent with the trends shown in Figure 8c. Specifically, the PPO activities of the CK group and the PVA/CS group reached the maximum at 7 days d and 14 days, respectively, and then decreased. In contrast, the PPO activity of the PVA/CS/ACP/TTEO group increased slowly throughout the shelf life and was always significantly lower than that of the control group. This difference was attributed to the antioxidant capacity of the film and its barrier effect, which prevented oxygen from contacting the phenolic substances in the fruit. The mechanism is in line with the findings of Xing et al. [112]. Their study fabricated a chitosan/nano-titanium dioxide film on mangoes to create a stable microenvironment, efficiently suppressing the PPO activity and reducing the fruit MDA content.

#### 3.2.4. Contents of H_2_O_2_ and MDA

H_2_O_2_ is a type of ROS, and its content change reflects the oxidative stress status of fruits. Figure 8d shows that the H_2_O_2_ content in the CK group increased sharply at 14 days, while the accumulation rate of H_2_O_2_ in the other groups was slower, and, at 21 days, the H_2_O_2_ content in the PVA/CS/ACP/TTEO group was significantly lower compared with the other two groups. Previous studies [113] found that PVA-based films incorporated with peppermint extract containing peppermint essential oil could slow down the accumulation of H_2_O_2_ in sweet peppers and maintain ROS homeostasis. MDA is an indicator for evaluating the degree of cell damage, and an increase in its content indicates a damaged membrane structure [114]. Figure 8e shows that, compared with the CK group, the increase in MDA content in both the PVA/CS group and the PVA/CS/ACP/TTEO group was smaller, with the latter being the lowest. This indicates that the film helps reduce H_2_O_2_ production, maintain high activities of SOD and CAT in fruits to enhance H_2_O_2_ scavenging, reduce membrane lipid peroxidation, and inhibit MDA accumulation. Another study [115] reported that clove essential oil could inhibit membrane lipid oxidation in yellow peach flesh tissue, and its incorporation into CS/PVA packaging could reduce the accumulation rate of MDA in yellow peaches at room temperature.

#### 3.2.5. Relative Conductivity

Relative conductivity is commonly used to characterize cell membrane permeability, and its variation can reflect the integrity and stability of cell membranes, which is one of the key indicators for indirectly evaluating the cell membrane integrity of fruits and vegetables [116]. Figure 8f shows that the relative conductivity of all groups exhibited a gradual upward trend during their shelf life. No significant differences were detected among the treatment groups at 7 days. At 21 days, the relative conductivity increased sharply in all groups, while that of the PVA/CS/ACP/TTEO group showed significantly lower values than the other two groups. This difference was attributed to the lower water vapor and AOP of the film, which may reduce the damage caused by the external environment to the cell membrane. Dai et al. [117] also demonstrated that nanocomposite coatings reduced the cell membrane permeability and PPO activity of Huangguan pears during storage.

#### 3.2.6. Lignin Content

Lignin is a major structural component of plant cell walls, and its biosynthesis is regulated not only during development but also induced under stresses such as wounding, UV irradiation, and pathogen attack [118]. As shown in Figure 8g, the lignin content in all groups first increased and then decreased. This was attributed to the activation of lignin synthesis in the early stage to enhance fruit resistance and adapt to environmental changes, while, in the later stage, the physiological function of fruits declined, the activity of synthetic enzymes decreased, and the degradation mechanism might be initiated to maintain vital activities. At 7 days of storage, the lignin content of the PVA/CS/ACP/TTEO group was significantly lower than that of the CK group, and no significant differences were observed among the groups thereafter. The composite film may alleviate the cell damage caused by early oxidative stress, and the stable microenvironment constructed by the film inhibits the activity of enzymes related to lignin synthesis.

### 3.3. Microbial Analysis

**Figure 9:**
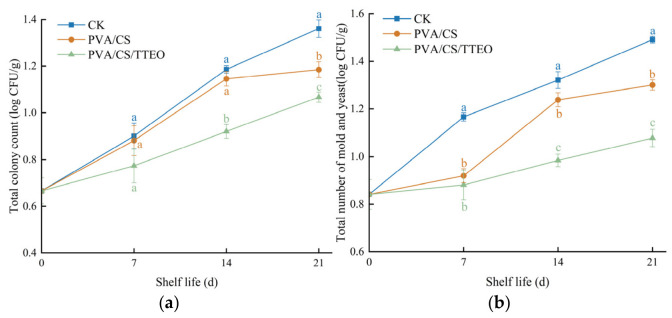
Effects of different treatments on microorganisms in ‘Yuluxiang’ pear fruits during shelf life: (**a**) changes in the total number of colonies; and (**b**) changes in the number of molds and yeasts. Distinct letters for the same index at each 7-day interval represent significant differences between groups (*p* < 0.05).

Microbial infection is primary factor responsible for the deterioration of fruits and vegetables, and fruits become increasingly susceptible to infection during ripening [119]. As presented in Figure 9a,b, compared with the CK group, PVA/CS film exerted a certain inhibitory effect on microbial growth in pears, which was attributed to the inherent antibacterial activity of CS and the barrier property of the film. However, its inhibitory effect gradually weakened with the extension in storage time. The PVA/CS/ACP/TTEO film-treated group showed no significant difference in bacterial count compared with the control group at 7 days of storage, and significantly delayed microbial growth in pears at 14 and 21 days. This might be attributed to the synergistic effect of CS and TTEO, which destroys the bacterial cell structure, interferes with their physiological functions, and simultaneously inhibits the growth of molds and the division of yeasts. These findings are consistent with a previous study [120], which reported that polyvinyl alcohol/CS-based films containing TTEO could effectively inhibit the increase in the microbial count in red grapes.

## 4. Conclusions

The present study demonstrates that the PVA/CS-based composite film functionalized with ACP and TTEO exhibits strong potential as a natural active packaging material for extending the room-temperature shelf life of ‘Yuluxiang’ pears. ACP and TTEO are stably bound to the film matrix through intermolecular interactions, optimizing the mechanical properties, barrier characteristics, and antioxidant and antibacterial activities of the composite film. In particular, the composite film effectively regulates the permeation of water vapor and oxygen. During the 21-days storage period, ‘Yuluxiang’ pears treated with the PVA/CS/ACP/TTEO composite film exhibited excellent quality retention, including reduced fruit weight loss and microbial spoilage, as well as well-maintained firmness, color, and key nutritional components, such as vitamin C and titratable acids. Moreover, by regulating the antioxidant enzyme system and alleviating the membrane lipid peroxidation damage, the composite film delays fruit senescence and the occurrence of peel greasiness and yellowing. Nevertheless, this study has certain limitations: the trace migration of TTEO from composite films may slightly affect the sensory aroma of fruits during storage, and the safety and relevant regulatory properties of ACP and TTEO for food contact require further investigation. Furthermore, it is also necessary to explore how to ensure that the films maintain structural integrity and normal functions when applied for the preservation of pears and other fruits. Overall, these findings indicate that the co-incorporation of ACP and TTEO into composite film contributes to an improved fruit preservation efficiency, supports the practical applicability of ACP in food packaging, and provides an eco-friendly, feasible technical approach for the room-temperature preservation of perishable fruits and vegetables, such as ‘Yuluxiang’ pears.

## Figures and Tables

**Table 1:** Color parameters (L*, a*, b*, and ΔE) and opacity of the film.

Indicators	PVA/CS	PVA/CS/ACP	PVA/CS/ACP/TTEO
Opacity	0.46 ± 0.01 ^c^	0.50 ± 0.01 ^a^	0.49 ± 0.01 ^b^
L*	56.89 ± 0.84 ^a^	47.39 ± 2.21 ^b^	49.17 ± 1.89 ^b^
a*	−0.61 ± 0.03 ^c^	0.05 ± 0.11 ^a^	−0.39 ± 0.10 ^c^
b*	0.03 ± 0.12 ^c^	10.22 ± 1.19 ^a^	8.18 ± 0.11 ^b^
ΔE	5.21 ± 0.82 ^b^	46.41 ± 0.82 ^a^	44.12 ± 1.84 ^a^

Means with different superscript letters within the same row differ significantly (*p* < 0.05).

**Table 2:** Performance parameters of PVA/CS, PVA/CS/ACP, and PVA/CS/ACP/TTEO.

Film Properties	PVA/CS	PVA/CS/ACP	PVA/CS/ACP/TTEO
Thickness (mm)	0.150 ± 0.002 ^a^	0.154 ± 0.002 ^a^	0.155 ± 0.003 ^a^
WC (%)	69.22 ± 0.45 ^a^	56.43 ± 2.77 ^b^	51.58 ± 1.43 ^b^
SD (%)	1074.79 ± 85.14 ^a^	666.34 ± 82.74 ^b^	594.69 ± 82.29 ^b^
WS (%)	7.60 ± 0.36 ^b^	41.26 ± 3.64 ^a^	41.34 ± 1.69 ^a^
WVP (×10^−10^ g·m^−1^·s^−1^·Pa^−1^)	3.46 ± 0.01 ^b^	3.82 ± 0.23 ^a^	3.16 ± 0.01 ^c^
AOP (mg·cm^−2^·d^−1^)	2.16 ± 0.06 ^b^	2.39 ± 0.06 ^a^	1.94 ± 0.12 ^c^
TS (MPa)	3.88 ± 0.04 ^b^	4.05 ± 0.06 ^a^	4.13 ± 0.05 ^a^
EAB (%)	378.00 ± 3.73 ^b^	392.21 ± 3.12 ^a^	382.22 ± 4.06 ^b^

Means with different superscript letters within the same row differ significantly (*p* < 0.05).

**Table 3:** Changes in peel color of ‘Yuluxiang’ pears under different treatments.

Peel Color Parameters	Shelf Life (Day)	CK	PVA/CS	PVA/CS/ACP/TTEO
L*	0	67.09 ± 0.10 ^a^	67.09 ± 0.10 ^a^	67.09 ± 0.10 ^a^
	7	67.11 ± 0.05 ^b^	67.46 ± 0.18 ^a^	67.02 ± 0.22 ^b^
	14	68.90 ± 0.15 ^a^	66.76 ± 0.19 ^c^	67.27 ± 0.03 ^b^
	21	70.41 ± 0.10 ^a^	66.10 ± 0.29 ^c^	67.28 ± 0.11 ^b^
a*	0	−3.38 ± 0.11 ^a^	−3.38 ± 0.11 ^a^	−3.38 ± 0.11 ^a^
	7	−1.42 ± 0.07 ^a^	−2.29 ± 0.05 ^b^	−2.7 ± 0.03 ^c^
	14	1.02 ± 0.02 ^a^	−1.59 ± 0.02 ^b^	−2.22 ± 0.04 ^c^
	21	3.53 ± 0.12 ^a^	−0.41 ± 0.08 ^b^	−1.09 ± 0.10 ^c^
b*	0	43.26 ± 0.21 ^a^	43.26 ± 0.21 ^a^	43.26 ± 0.21 ^a^
	7	46.58 ± 0.08 ^a^	46.43 ± 0.09 ^a^	45.49 ± 0.25 ^b^
	14	48.94 ± 0.16 ^a^	47.67 ± 0.21 ^b^	46.96 ± 0.11 ^c^
	21	51.13 ± 0.07 ^a^	48.03 ± 0.17 ^b^	47.88 ± 0.07 ^b^

Means with different superscript letters within the same line indicate significant differences (*p* < 0.05).

**Table 4:** Influence of different treatments on fruit quality parameters of pears during shelf life.

Shelf Life (Day)	Films	Firmness (N)	TSS (%)	TA (%)	Vitamin C (mg/100 g)	Weight Loss (%)
0	-	21.70 ± 0.10	11.37 ± 0.06	0.26 ± 0.021	15.72 ± 0.11	-
7	CK	20.15 ± 0.15 ^b^	12.20 ± 0.10 ^a^	0.18 ± 0.012 ^b^	13.26 ± 0.11 ^b^	2.47 ± 0.37 ^a^
	PVA/CS	21.03 ± 0.10 ^a^	11.73 ± 0.12 ^b^	0.23 ± 0.020 ^a^	13.39 ± 0.19 ^b^	1.98 ± 0.38 ^b^
	PVA/CS/ACP/TTEO	21.30 ± 0.14 ^a^	11.60 ± 0.10 ^b^	0.24 ± 0.009 ^a^	14.84 ± 0.11 ^a^	1.86 ± 0.18 ^b^
14	CK	19.31 ± 0.16 ^b^	12.23 ± 0.06 ^a^	0.19 ± 0.017 ^b^	13.20 ± 0.19 ^b^	4.15 ± 0.66 ^a^
	PVA/CS	19.63 ± 0.20 ^b^	11.90 ± 0.17 ^a^	0.20 ± 0.010 ^b^	13.20 ± 0.38 ^b^	4.13 ± 0.65 ^a^
	PVA/CS/ACP/TTEO	20.22 ± 0.26 ^a^	11.83 ± 0.32 ^a^	0.24 ± 0.011 ^a^	14.78 ± 0.55 ^a^	4.14 ± 0.33 ^a^
21	CK	18.30 ± 0.12 ^b^	12.40 ± 0.35 ^a^	0.16 ± 0.011 ^b^	10.55 ± 0.33 ^c^	7.08 ± 0.43 ^a^
	PVA/CS	18.28 ± 0.20 ^b^	12.27 ± 0.25 ^a^	0.18 ± 0.021 ^ab^	12.50 ± 0.29 ^b^	6.91 ± 0.68 ^ab^
	PVA/CS/ACP/TTEO	19.20 ± 0.43 ^a^	12.23 ± 0.21 ^a^	0.20 ± 0.010 ^a^	13.58 ± 0.33 ^a^	6.55 ± 0.31 ^b^

Different letters for the same index at each 7-day sampling point indicate significant differences among groups (*p* < 0.05).

## Data Availability

The original contributions presented in this study are included in the article. Further inquiries can be directed to the corresponding authors.
